# CompoundHetVIP: Compound Heterozygous Variant Identification Pipeline

**DOI:** 10.12688/f1000research.26848.2

**Published:** 2021-02-10

**Authors:** Dustin B. Miller, Stephen R. Piccolo

**Affiliations:** 1 Department of Biology, Brigham Young University, Provo, UT, 84602, USA

**Keywords:** Genetics, compound heterozygous, genome analysis, trio, phasing, reproducibility

## Abstract

Compound Heterozygous ( *CH*) **variant identification requires distinguishing maternally from paternally derived nucleotides, a process that requires numerous computational tools. Using such tools often introduces unforeseen challenges such as installation procedures that are operating-system specific, software dependencies that must be installed, and formatting requirements for input files. To overcome these challenges, we developed Compound Heterozygous Variant Identification Pipeline (CompoundHetVIP), which uses a single Docker image to encapsulate commonly used software tools for file aggregation ( *BCFtools *or *GATK4*), VCF liftover ( *Picard Tools*), joint-genotyping ( *GATK4*), file conversion ( *Plink2*), phasing ( *SHAPEIT2*, *Beagle*, and/or *Eagle2*), variant normalization ( *vt *tools), annotation ( *SnpEff*), relational database generation ( *GEMINI*), and identification of *CH*, homozygous alternate, and *de novo* variants in a series of 13 steps. To begin using our tool, researchers need only install the Docker engine and download the CompoundHetVIP Docker image. The tools provided in CompoundHetVIP, subject to the limitations of the underlying software, can be applied to whole-genome, whole-exome, or targeted exome sequencing data of individual samples or trios (a child and both parents), using VCF or gVCF files as initial input. Each step of the pipeline produces an analysis-ready output file that can be further evaluated. To illustrate its use, we applied CompoundHetVIP to data from a publicly available Ashkenazim trio and identified two genes with a candidate *CH *variant and two genes with a candidate homozygous alternate variant after filtering based on user-set thresholds for global minor allele frequency, Combined Annotation Dependent Depletion, and Gene Damage Index. While this example uses genomic data from a healthy child, we anticipate that most researchers will use CompoundHetVIP to uncover missing heritability in human diseases and other phenotypes. CompoundHetVIP is open-source software and can be found at https://github.com/dmiller903/CompoundHetVIP; this repository also provides detailed, step-by-step examples.

## Introduction

A compound heterozygous ( *CH*) variant occurs when a person inherits two alleles, one from each parent, and these alleles are located at different positions within the same gene ^1^. The compound effects of these alternate alleles may lead to phenotypic effects as seen in some cases of human disease, including ataxia telangiectasia, NGLY1 deficiency, and various types of pediatric cancer ^2– 4^. For example, *CH* variants in the mismatch repair gene, *MSH6*, have been identified in pediatric patients with colorectal cancer, medulloblastoma, high-grade glioma, glioblastoma, non-Hodgkin’s lymphoma, and acute lymphoblastic leukemia ^4^. To detect *CH* variants in next-generation sequencing data, it is necessary to differentiate between paternally and maternally derived nucleotides ^1^. Laboratory-based methods such as fosmid-pool-based or linked-read sequencing can be used; however, if DNA libraries are prepared and sequenced without regard to nucleotide inheritance (as is done in most sequencing projects), computational methods can help determine parental inheritance through haplotype estimation (“phasing”) ^5– 7^.

Available phasing tools require specific input file types (such as VCF or *Plink* files) and reference files which are not standardized across different phasing software. In addition, many phasing programs require that input files have been aligned to a specific reference genome, do not contain multiallelic positions, are free of repeat positions, and that each chromosome be phased separately ^8– 10^. Figuring out how to prepare files for phasing can be challenging as passing files from program to program may result in unforeseen incompatibilities. Additionally, installing some programs can be challenging because of operating-system specific installation processes and software dependencies.

We have designed Compound Heterozygous Variant Identification Pipeline (CompoundHetVIP) to help researchers overcome these time-consuming challenges when identifying *CH* variants. CompoundHetVIP encapsulates specific versions of existing tools, required software dependencies, and custom Python scripts into a cohesive computational environment packaged as a Docker image ^11^. Accordingly, researchers need only install the Docker software and download the CompoundHetVIP Docker image to begin performing *CH*, homozygous alternate, and *de novo* analyses at the command line. Furthermore, because the source code for CompoundHetVIP is publicly available, other researchers will be able to reproduce the analyses and investigate the specific methodologies used.

## Methods

### Implementation

**Figure 1.**
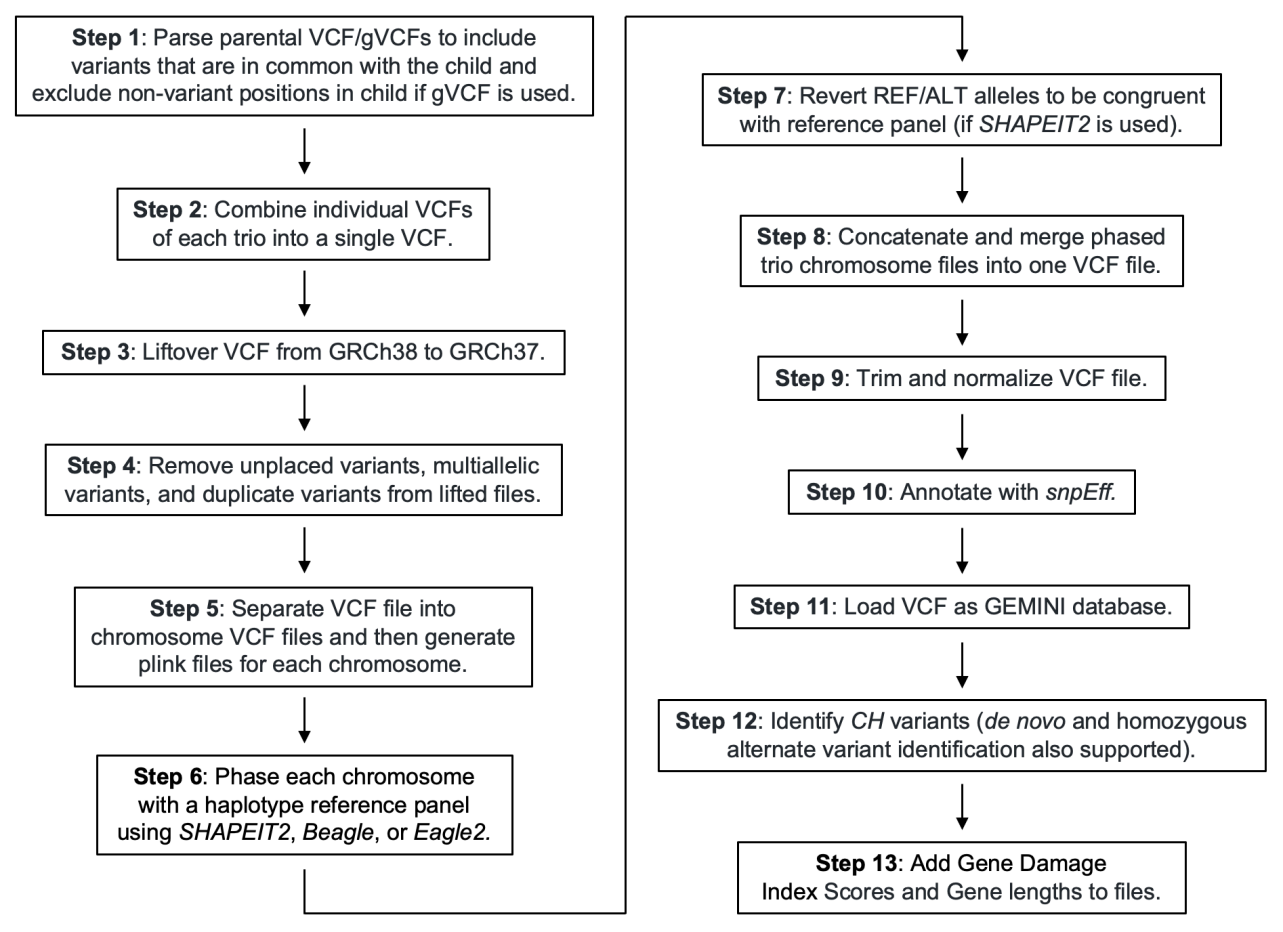
Flow diagram of CompoundHetVIP functionality.

The functionality of CompoundHetVIP is divided into a series of 13 steps ( Figure 1). For each step, a Python script is executed within a Docker container. These scripts provide logic for processing data files and invoking third-party tools. By breaking the pipeline into 13 steps, users have flexibility to perform the steps that are most relevant to their analysis. For example, researchers can use input data for an affected individual only or for a trio (an affected individual and both parents). If parental data are unavailable and the variant positions within the VCF file correspond to genome build GRCh37, users may skip the first three steps. A detailed, step-by-step guide is available on GitHub and as *Extended data* ^12^.

### Workflow

For **step 1**, the inputs can be either Variant Call Format (VCF) ^13^ or gVCF ^14^ files that were generated from whole-genome, whole-exome, or targeted exome sequencing data. VCF files contain variant sites only, whereas gVCF files include non-variant sites, too. For each parent of a trio being evaluated, our script retains nucleotide positions that are in common with the child. When gVCF files are used (whether for trios or individuals), our script removes all non-variant sites for the child (but retains these for the parents to support determination of *CH* status). When applied to trio data, some phasing tools, such as *SHAPEIT2* ^8^, require a single input file for each trio. Therefore, in **step 2** (used only when working with trios), we combine the variant files for each member of a trio into a single VCF file using either *BCFtools* (VCF input files) ^15^ or *GATK4* (gVCF input files) ^14^. If *GATK4* is used, joint-genotyping is also performed on the trio VCF.

The remaining steps can be applied either to trios or individuals. Some phasing and annotation programs require that data be aligned to genome build GRCh37; thus, we use this reference genome as our standard. For variant files that have been aligned to genome build GRCh38, **step 3** uses *Picard Tools* ^16^ to convert the data to GRCh37 positions using a lift-over process. During lift-over, some sites may be present in GRCh38, but their exact position in GRCh37 is unknown. To avoid ambiguity, these sites are removed during **step 4**. This step also removes positions that are multiallelic or duplicated to maintain compatibility with programs such as *Plink2* ^17, 18^ and *SHAPEIT2* (used in steps 5 and 6, respectively). For trio VCF files, sites that contain missing genotype information (i.e. “./.”) for both parents are removed to improve phasing accuracy.

CompoundHetVIP can perform phasing using *SHAPEIT2*, *Eagle2* ^10^, and/or *Beagle* ^9^. Each of these programs requires that each chromosome be phased independently. Additionally, when using *SHAPEIT2,* it is recommended that PLINK files (.bed, .bim, .fam) be used as input for phasing. Therefore, **step 5** divides a VCF file by chromosome into multiple files and creates the necessary PLINK files for each chromosome (when SHAPEIT2 is used for phasing).

**Step 6** phases the variants in each chromosome using default parameters for the phasing program chosen by the user. We recommend using *SHAPEIT2* because it can applied either to trios or individuals. When parents’ genotypes are available, this program uses Mendelian logic for phasing and a population-based haplotyped reference panel when the phase of the child cannot be determined from Mendelian logic alone (i.e. both parents and child are heterozygous). In addition, if a parent is missing genotype information at a position, *SHAPEIT2* imputes the missing information. All supported phasing programs integrate the 1000 Genomes Project phase 3 haplotype reference panel ^19^ and do not require sequence alignment files (.bam), such as those required by read-based programs ^20, 21^. In some scenarios, *SHAPEIT2* switches the REF and ALT alleles. Therefore, **step 7** ensures that the REF/ALT alleles of the phased VCF files are congruent with those of the reference genome. Also, sites with Mendelian errors are removed.

To make subsequent analysis of the phased files easier, **step 8** concatenates all phased chromosomes into a single file. If a user is analyzing multiple trios (or individuals), this script can also merge the data for these trios (or individuals) into a single VCF file.

**Step 9** normalizes VCF files as recommended by *GEMINI* ^22^ (used in step 11). Normalization involves left-alignment and trimming of variants ^23^. This process helps ensure that variants are represented at their left-most position, with as few nucleotides as possible, and unambiguously. This step uses *vt tools* ^23^. In **step 10,** *SnpEf* ^24^ provides information about the effects of variants on function for known genes. Then, in **step 11**, *GEMINI* ^22^ loads the annotated VCF into a relational database ( *GEMINI* can also load files annotated with Variant Effect Predictor ( *VEP*) ^25^, although *VEP* is not available as part of our pipeline). **Step 12** uses a custom Python script to extract *CH* variant data from the database. Our provided script identifies *CH* variants and filters the data based on user-set thresholds for global minor allele frequency (MAF) and Combined Annotation Dependent Depletion (CADD) scores ^26^. Variants with a MAF less than or equal to the user-set threshold, CADD score greater than or equal to the user-set threshold, exonic classification, and “HIGH” or “MED” putative impact severity are included in the final output. We consider the variants in the final output as candidates for further evaluation. For step 12, we provide two additional scripts that identify homozygous alternate variants and *de novo* variants using the same user-set thresholds as those described above.

Finally, in **step 13**, we add Gene Damage Index (GDI) scores ^27^ and gene-length information to the output files. GDI scores quantify accumulated mutational damage in healthy populations as a way to predict whether genes are likely to have disease-causing variants. Genes of longer length (e.g. *TTN*, *MUC5B*) tend to have more total damage but typically less disease-causing damage than shorter genes.

### Operation

Because CompoundHetVIP executes all scripts within a Docker container, it can be executed on all major operating systems that are commonly used for scientific computing. Depending on input file sizes, the hardware needed to execute CompoundHetVIP will vary from user to user. Certain tasks, such as phasing (step 6), can be memory intensive. A minimum of 40 GB memory is recommended. When creating a relational database with *GEMINI* (step 11), there is no minimum processing core recommendation, but multiprocessing can significantly speed up the time it takes to load the database. Users can specify how many processing cores *GEMINI* can use when executing step 11.

## Results

We applied CompoundHetVIP to high-confidence, VCF data that were generated with whole-genome sequencing data from an Ashkenazim trio available through the Genome in a Bottle Consortium ^28^. During step 6, we used *SHAPEIT2* to phase the data. In the child of this trio, we identified a *CH* variant in two genes ( *FLNB* and *TTN*) using a MAF threshold of 0.01 and a CADD score threshold of 15. Genes with a GDI score less than or equal to 13.84 are classified as being more likely to have disease-causing damage from variants ^27^. *FLNB* (6.2) was lower than this threshold but *TTN* (42.9) was not. *FLNB* has an important role in cytoskeleton development and variations in this gene have been associated with many skeletal disorders ^29, 30^.

In addition, we identified two homozygous alternate variants: one in *TBC1D2* and the other in *TOX2*, using the same MAF and CADD thresholds that we used for *CH* variant identification. *TBC1D2* and *TOX2* had GDI scores of 9.7 and 4.4, respectively. *TBC1D2* codes for a GTPase-activating protein and is involved in E-cadherin degradation ^31^. The role of this gene and how it may relate to human disease is not yet fully understood. *TOX2* is a transcription factor that helps drive the development of T follicular helper (Tfh) cells ^32^. Tfh cells are an important part of humoral immunity.

Using the same MAF and CADD thresholds described above, we sought to identify *de novo* variants in this trio. However, none passed these thresholds.

## Conclusion

CompoundHetVIP provides the necessary tools for *CH* variant identification using VCF or gVCF files as initial input and is executed within a Docker container, which allows for cross-platform compatibility and reproducibility. CompoundHetVIP involves 13 steps ( Figure 1) that include a breadth of tasks such as file aggregation, VCF liftover, joint-genotyping, file conversion, phasing, variant normalization, annotating, and variant identification. Our results highlight that potentially damaging *CH* and homozygous alternate variants are observed in seemingly healthy individuals. However, we anticipate that most researchers will use CompoundHetVIP to identify variants in individuals with a known disease.

## Data availability

### Source data

VCF data used to generate the results were from an Ashkenazim trio, freely-available through the Genome in a Bottle Consortium at ftp://ftp-trace.ncbi.nlm.nih.gov/giab/ftp/release/AshkenazimTrio/ ^27^:

- Child: ftp://ftp-trace.ncbi.nlm.nih.gov/giab/ftp/release/AshkenazimTrio/HG002_NA24385_son/latest/GRCh38/supplementaryFiles/HG002_GRCh38_CHROM1-22_v4.1_highconf.vcf.gz
- Mother: ftp://ftp-trace.ncbi.nlm.nih.gov/giab/ftp/release/AshkenazimTrio/HG004_NA24143_mother/latest/GRCh38/HG004_GRCh38_GIAB_highconf_CG-Illfb-IllsentieonHC-Ion-10XsentieonHC_CHROM1-22_v.3.3.2_highconf.vcf.gz
- Father: ftp://ftp-trace.ncbi.nlm.nih.gov/giab/ftp/release/AshkenazimTrio/HG003_NA24149_father/latest/GRCh38/HG003_GRCh38_GIAB_highconf_CG-Illfb-IllsentieonHC-Ion-10XsentieonHC_CHROM1-22_v.3.3.2_highconf.vcf.gz

### Extended data

Zenodo: dmiller903/CompoundHetVIP: CompoundHetVIP - v1.1. https://doi.org/10.5281/zenodo.4477686 ^12^.

This project contains the following extended data:

- CompoundHetVIP_example.pdf (detailed step-by-step example)

## Software availability

Software available from: https://hub.docker.com/r/dmill903/compound-het-vip

Source code available from: https://github.com/dmiller903/CompoundHetVIP

Archived source code at time of publication: https://doi.org/10.5281/zenodo.4477686 ^12^.

License: MIT
