# Supplementary material for: CompoundHetVIP: Compound Heterozygous Variant Identification Pipeline
Source: F1000Res. 2021 Feb 10;9:1211. Originally published 2020 Oct 8. [Version 2] doi: 10.12688/f1000research.26848.2 (PMC7905494; doi:10.12688/f1000research.26848.2)
Supplement: Peer review history

# Peer review: Reviewer response for version 2

**DOI:** 10.5256/f1000research.54440.r79307

**Published:** 2021-02-24

**Authors:** Soohyun Lee

**Affiliations:** 1 Department of Biomedical Informatics, Harvard Medical School, Boston, Massachusetts, USA

I thank the authors for addressing all my comments and I have no further comments to make.

Are the conclusions about the tool and its performance adequately supported by the findings presented in the article?

Partly

Is the rationale for developing the new software tool clearly explained?

No

Is the description of the software tool technically sound?

Yes

Are sufficient details of the code, methods and analysis (if applicable) provided to allow replication of the software development and its use by others?

Partly

Is sufficient information provided to allow interpretation of the expected output datasets and any results generated using the tool?

Partly

Reviewer Expertise:

genomics, transcriptomics, genomic variant calling and analysis, software and algorithm development, cloud infrastructure, docker, genomic pipeline

I confirm that I have read this submission and believe that I have an appropriate level of expertise to confirm that it is of an acceptable scientific standard.

# Peer review: Reviewer response for version 1

**DOI:** 10.5256/f1000research.29647.r76628

**Published:** 2021-01-25

**Authors:** Sushant Patil

**Affiliations:** 1 Department of Pathology and Laboratory Medicine, University of North Carolina, Chapel Hill, North Carolina, USA

Thank you for the opportunity to review the manuscript "CompoundHetVIP: Compound Heterozygous Variant Identification Pipeline" by Miller *et al.*

 I find the published software to be of great utility, even though it may not have scientific novelty as such. The manuscript is neat and well-presented. I recommend this manuscript for publication in the journal. However, I request the authors to address the following points in the final revision:

1. It appears that the program can only be used with whole-genome sequencing VCFs. Is that true? How about VCFs generated from WES or targeted panel?
2. Step 5 - "CompoundHetVIP can perform phasing using SHAPEIT2, Eagle210, and/or Beagle9.". Is there a 'default' mode of execution for the software? If not, I suggest to have one and include one or all of these phasing programs as part of it. 
3. In Figure 1, I recommend labeling each box with the corresponding Step # to relate it to the text in the manuscript. 
4. Ideally all VCFs should be 1-based. In a hypothetical scenario, if the parents' VCFs are 0-based and the child's 1-based (coming from different workflows), will the program be able to detect and handle such discrepancy? 
5. I recommend saying minor allele frequency (MAF) as Global minor allele frequency (MAF) at first instance. It is easy to be mistaken that for mutant allelic frequency in cancer genomics. 

Are the conclusions about the tool and its performance adequately supported by the findings presented in the article?

Partly

Is the rationale for developing the new software tool clearly explained?

Yes

Is the description of the software tool technically sound?

Yes

Are sufficient details of the code, methods and analysis (if applicable) provided to allow replication of the software development and its use by others?

Yes

Is sufficient information provided to allow interpretation of the expected output datasets and any results generated using the tool?

Yes

Reviewer Expertise:

Bioinformatics, Computational Biology, NGS/HTS, Cancer/Oncology, Personalized Medicine

I confirm that I have read this submission and believe that I have an appropriate level of expertise to confirm that it is of an acceptable scientific standard.

# Response

**Published:** 2021-02-02

**Authors:** Dustin Miller

**Affiliations:** Brigham Young University, USA

Thank you for taking the time to review our manuscript. We appreciate your thorough and helpful comments. We have addressed these comments below in a point-by-point response. These changes have made the manuscript more informative and insightful.

 The reviewers comments are in regular font. Our responses are in **bold**.

 1. It appears that the program can only be used with whole-genome sequencing VCFs. Is that true? How about VCFs generated from WES or targeted panel?

**Thank you for pointing this out. We designed this pipeline with WGS in mind and did not think about other types of next-gen data. To test the pipelines on WES/targeted exome data, we ran the pipeline on a publicly available dataset available here: https://my.pgp-hms.org/profile/huF85C76. The data we used was the “Helix Exome+ WES” VCF file ( https://my.pgp-hms.org/user_file/download/3888). After filtering the data for positions that passed quality filters and had a quality > 20, we started on step 3 and went through all remaining steps. The pipeline worked well. We have made this more clear in the manuscript.**

 2. Step 5 - "CompoundHetVIP can perform phasing using SHAPEIT2, Eagle210, and/or Beagle9.". Is there a 'default' mode of execution for the software? If not, I suggest to have one and include one or all of these phasing programs as part of it. 

**We have a separate script for all 3 phasing programs. That way the user can choose which program to use. Within each script, the phasing software is executed under default parameters. Our example PDF uses SHAPEIT2 and we recommend this as the default phaser to use, especially when using trio data. However, we feel that the choice of phasing program is fundamental to our pipeline and should require an explicit decision from the user; so we require the user to select one. We have made this more clear in the example documentation ( https://github.com/dmiller903/CompoundHetVIP/blob/master/CompoundHetVIP_example.pdf) and in the manuscript.**

 3. In Figure 1, I recommend labeling each box with the corresponding Step # to relate it to the text in the manuscript. 

**Thank you for the recommendation. We have updated the figure to show step numbers.**

 4. Ideally all VCFs should be 1-based. In a hypothetical scenario, if the parents' VCFs are 0-based and the child's 1-based (coming from different workflows), will the program be able to detect and handle such discrepancy? 

**This pipeline assumes input VCF files are 1-based. We added this disclaimer to the example documentation referenced in response 2. During step 11, GEMINI 0-bases the positions. However, during step 12, they are reverted back to 1-based positions for consistency.**

 5. I recommend saying minor allele frequency (MAF) as Global minor allele frequency (MAF) at first instance. It is easy to be mistaken that for mutant allelic frequency in cancer genomics.

**Thank you for this recommendation. We have updated the manuscript to say “global minor allele frequency” when first referenced.**

**In addition to the changes mentioned above, we have made a few other changes we would like to mention here:**

 1. We changed the way we filter the variants to be more simple but also more strict. Originally we allowed some variants to be considered if they were of “HIGH” putative impact but were missing CADD or MAF values. To obtain the variants most likely to play a role in disease, we made the requirement more strict. Now, CADD and MAF scores must be available for all variants, variants must be in exonic regions, and variants can have either “MED” or “HIGH” putative impact. Making these changes did not change the number of CH variants identified in the example dataset, but will likely have an effect in other datasets. This change in how variants are filtered may provide the users with a smaller subset of putative variants, but the CompoundHetVIP will provide more information for the users to use in downstream analysis (i.e. CADD and MAF values for all variants rather than just some of the variants). Our description of the filtering method has been updated in the manuscript. 

 2. There was an error in our script that identified homozygous alternate variants. Some of our previous code required that a gene have 2 variants in order to be written to the output file. This has been fixed, and it slightly changed the results. Originally we reported only 1 gene with a homozygous alternate variant in the example dataset. After updating the script and running it again, there are 2 genes that have a homozygous alternate variant. This result has been modified in the manuscript.

 3. Minor updates to the example PDF ( https://github.com/dmiller903/CompoundHetVIP/blob/master/CompoundHetVIP_example.pdf) were made for added clarity and explanation.

# Peer review: Reviewer response for version 1

**DOI:** 10.5256/f1000research.29647.r76619

**Published:** 2021-01-20

**Authors:** Soohyun Lee

**Affiliations:** 1 Department of Biomedical Informatics, Harvard Medical School, Boston, Massachusetts, USA

The paper describes a software pipeline that identifies compound heterozygous (CH) variants from the VCF or gVCF files of whole genome sequencing data from either a single sample or a trio. The pipeline has 13 steps and in brief, performs VCF file merging, liftover from hg38 to hg37, haplotype phasing, variant annotation and filtering based on minor allele frequency (MAF) and Combined Annotation Dependent Depletion (CADD) scores. The pipeline is dockerized for portable and reproducible analysis.

 The paper addresses an important problem of identifying CH variants. Many researchers seem to end up writing their own custom CH variant detecting tools and it could be a useful addition if that can be avoided.

 I have a few comments which hopefully would improve the clarity of the article and the usability of the pipeline. Before we get to that, I think I have to disclose that I myself and my team member designed and wrote our own CH variant caller as a part of our own variant calling/analysis pipeline, with the help of several experts in the genetics field (not necessarily for publication but for our own use). Therefore, my review is based on my experience tackling a similar problem but most likely with a different set of requirements/constraints.

1. For better clarity and flow of the article, it may be helpful to describe relatively early on (e.g. in the title or early in the abstract) what the pipeline does, including the data type (e.g. whole genome sequencing) and starting point (e.g. VCF/gVCF). The steps the pipeline offers could also be mentioned earlier. For example, it is not clear until near the end of the article that filtering based on MAF and CADD is performed as a part of the pipeline, and one could wonder simply reading the abstract, why only two genes were identified to have CH variants, which is much fewer than expected from a whole genome sequencing data. It is also not clear in the beginning that variant annotation and an optional GATK4-based variant calling is part of the pipeline.
2. I think the pipeline usability could be improved if it accommodates previously merged and annotated VCF files. A user may start with a VCF that is annotated with their annotation tool of choice (e.g. VEP which is a more recent tool than SnpEff) that they may want to use for other analysis consistently in addition to CH variant detection. It would be useful to support such a use case. Alternatively, the user may have a pre-merged VCF file generated by GATK4 from joint variant calling with the parameters of their own choice. The pipeline’s VCF merging step performs GATK4-based variant calling which may make more sense to be done along with the other GATK4-based upstream steps rather than as part of the compound het detection step, though that can be a matter of preference. Having this kind of modularity could appeal more to bioinformaticians who are setting up a full variant calling and analysis pipeline and just need a CH detection module. It may also be computationally more efficient to first annotate variants with which genes they overlap with and remove intergenic variants before performing CH detection because CH variants by definition would only occur inside a gene.
3. The pipeline performs population-based haplotype phasing based on e.g. SHAPEIT2 to identify CH variants. It is not very convincing though how much this adds compared to simply scanning a VCF file once to collect all pairs of heterozygous variants in a gene whose parents’ genotypes indicate that one comes from the mother and the other from the father. The authors of the latest version of the phasing tool, SHAPEIT4, seems to have used Mendelian logics as the truth set to evaluate the performance of its phasing imputation, suggesting that when the parents’ genotypes are directly available, it maybe be more accurate to use those genotypes than using a population-based imputation. ^1^ It may be helpful for a proband-only case where the parents’ genotypes are not available. However, for a trio case, detecting CH variants from a previously annotated VCF file by simply comparing the genotypes of the parents would take one step of scanning the file (rather than 13 steps). I think having a more convincing rationale about why these additional steps are needed for a trio case would be very helpful.
4. I think the pipeline can definitely be useful as it is for specific users who do not have their own variant calling or annotation capacity and took an unannotated VCF or gVCF file from an external source and simply want to perform CH variant identification without parent information. Again, going back to point 1), having a full description of what the pipeline offers (including variant annotation, filtering, even running GATK4) in the title and early on in the abstract could make it easier to reach the right type of potential users.
5. Lastly a minor point, it would be useful to clarify if the pipeline also works for whole exome sequencing data.

Are the conclusions about the tool and its performance adequately supported by the findings presented in the article?

Partly

Is the rationale for developing the new software tool clearly explained?

No

Is the description of the software tool technically sound?

Yes

Are sufficient details of the code, methods and analysis (if applicable) provided to allow replication of the software development and its use by others?

Partly

Is sufficient information provided to allow interpretation of the expected output datasets and any results generated using the tool?

Partly

Reviewer Expertise:

genomics, transcriptomics, genomic variant calling and analysis, software and algorithm development, cloud infrastructure, docker, genomic pipeline

I confirm that I have read this submission and believe that I have an appropriate level of expertise to confirm that it is of an acceptable scientific standard, however I have significant reservations, as outlined above.

## References

[1] : Accurate, scalable and integrative haplotype estimation. *Nature Communications*.2019;10(1) : 10.1038/s41467-019-13225-y 10.1038/s41467-019-13225-y PMC688285731780650

# Response

**Published:** 2021-02-02

**Authors:** Dustin Miller

**Affiliations:** Brigham Young University, USA

Thank you for taking the time to review our manuscript. We appreciate your thorough and helpful comments. We have addressed these comments below in a point-by-point response. These changes have made the manuscript more informative and insightful.

 The reviewers comments are in regular font. Our responses are in **bold**.

 1. For better clarity and flow of the article, it may be helpful to describe relatively early on (e.g. in the title or early in the abstract) what the pipeline does, including the data type (e.g. whole genome sequencing) and starting point (e.g. VCF/gVCF). The steps the pipeline offers could also be mentioned earlier. For example, it is not clear until near the end of the article that filtering based on MAF and CADD is performed as a part of the pipeline, and one could wonder simply reading the abstract, why only two genes were identified to have CH variants, which is much fewer than expected from a whole genome sequencing data. It is also not clear in the beginning that variant annotation and an optional GATK4-based variant calling is part of the pipeline.

****

**Thank you for these recommendations. We have updated the abstract to get to the point more quickly, to mention the tools used for each major step, and to clarify that MAF and CADD are used as part of variant identification. We also updated the abstract to mention that whole-exome and targeted exome data can also be used. This updated abstract should provide a clearer understanding of what tools are included in the pipeline, and what data can be used as initial input.**

 2. I think the pipeline usability could be improved if it accommodates previously merged and annotated VCF files. A user may start with a VCF that is annotated with their annotation tool of choice (e.g. VEP which is a more recent tool than SnpEff) that they may want to use for other analysis consistently in addition to CH variant detection. It would be useful to support such a use case. Alternatively, the user may have a pre-merged VCF file generated by GATK4 from joint variant calling with the parameters of their own choice. The pipeline’s VCF merging step performs GATK4-based variant calling which may make more sense to be done along with the other GATK4-based upstream steps rather than as part of the compound het detection step, though that can be a matter of preference. Having this kind of modularity could appeal more to bioinformaticians who are setting up a full variant calling and analysis pipeline and just need a CH detection module. It may also be computationally more efficient to first annotate variants with which genes they overlap with and remove intergenic variants before performing CH detection because CH variants by definition would only occur inside a gene.

**Thank you for your thoughtful comments about the modularity of this pipeline. This is something we have thought about over the course of this project, and we want this pipeline to be flexible for diverse types of usage. We have updated the “gemini_load.py” script with an optional argument to allow users to import a file that was annotated with VEP. Because SnpEff is used as part of the pipeline, the script defaults to SnpEff; but if a user chooses to use VEP outside of the pipeline, they can still apply the remaining steps of the pipeline. Intergenic regions are not considered during the CH detection step. Within the “identify_CH_variants.py” script, the GEMINI database is converted to a TSV file; and during this process, intergenic regions are removed to speed up the CH detection process. Accordingly, it would be fine if researchers uploaded a VCF file from which intergenic regions have already been removed. In addition, we will be actively monitoring GitHub for any feature recommendations or any issues that arise. For users who are more experienced with coding and aren’t afraid of source code, the code is publicly available, and these users can adapt the code to meet their needs.**

****

 3. The pipeline performs population-based haplotype phasing based on e.g. SHAPEIT2 to identify CH variants. It is not very convincing though how much this adds compared to simply scanning a VCF file once to collect all pairs of heterozygous variants in a gene whose parents’ genotypes indicate that one comes from the mother and the other from the father. The authors of the latest version of the phasing tool, SHAPEIT4, seems to have used Mendelian logics as the truth set to evaluate the performance of its phasing imputation, suggesting that when the parents’ genotypes are directly available, it maybe be more accurate to use those genotypes than using a population-based imputation.1 It may be helpful for a proband-only case where the parents’ genotypes are not available. However, for a trio case, detecting CH variants from a previously annotated VCF file by simply comparing the genotypes of the parents would take one step of scanning the file (rather than 13 steps). I think having a more convincing rationale about why these additional steps are needed for a trio case would be very helpful.

**We considered coding an initial step that identifies all CH variants that can be inferred via Mendelian logic when both parents' genotypes are available. However, we wanted to make the pipeline consistent regardless of whether trio or individual data were used. Furthermore, SHAPEIT2 uses Mendelian logic when the parents' genotypes are available. The only time it uses compact hidden Markov model (CHMM) logic with trios is when the phase of the child cannot be determined using Mendelian logic alone (i.e. both parents are heterozygous, and the child is as well). Therefore, SHAPEIT2 provides the benefit of Mendelian logic and CHMM, when necessary. (The SHAPEIT4 paper also notes that it used the 500 trio children as validation only when they could be "phased using Mendel inheritance logic (i.e., no triple hets and no Mendel inconsistencies).") In addition, there may be scenarios where data is missing for one or both parents for a certain gene region, and population-based phasing with SHAPEIT2 has the added benefit of imputing the missing information. We have updated the manuscript to clarify these points, as suggested.**

****

 4. I think the pipeline can definitely be useful as it is for specific users who do not have their own variant calling or annotation capacity and took an unannotated VCF or gVCF file from an external source and simply want to perform CH variant identification without parent information. Again, going back to point 1), having a full description of what the pipeline offers (including variant annotation, filtering, even running GATK4) in the title and early on in the abstract could make it easier to reach the right type of potential users.

**Please see response # 1.**

****

 5. Lastly a minor point, it would be useful to clarify if the pipeline also works for whole exome sequencing data.

**Thank you for bringing this up. We designed this pipeline with WGS in mind, and did not think about other types of next-gen sequencing data. To test the pipelines on WES/targeted exome data, we ran the pipeline on a publicly available dataset available here: https://my.pgp-hms.org/profile/huF85C76. The data we used was the “Helix Exome+ WES” VCF file (https://my.pgp-hms.org/user_file/download/3888). After filtering the data for positions that passed quality filters and had a quality > 20, we started on step 3 and went through all remaining steps. The pipeline worked well. We have made this more clear in the manuscript.**

**In addition to the changes mentioned above, we have made a few other changes we would like to mention here:**

 1. We changed the way we filter the variants to be more simple but also more strict. Originally we allowed some variants to be considered if they were of “HIGH” putative impact but were missing CADD or MAF values. To obtain the variants most likely to play a role in disease, we made the requirement more strict. Now, CADD and MAF scores must be available for all variants, variants must be in exonic regions, and variants can have either “MED” or “HIGH” putative impact. Making these changes did not change the number of CH variants identified in the example dataset, but will likely have an effect in other datasets. This change in how variants are filtered may provide the users with a smaller subset of putative variants, but the CompoundHetVIP will provide more information for the users to use in downstream analysis (i.e. CADD and MAF values for all variants rather than just some of the variants). Our description of the filtering method has been updated in the manuscript. 

 2. There was an error in our script that identified homozygous alternate variants. Some of our previous code required that a gene have 2 variants in order to be written to the output file. This has been fixed, and it slightly changed the results. Originally we reported only 1 gene with a homozygous alternate variant in the example dataset. After updating the script and running it again, there are 2 genes that have a homozygous alternate variant. This result has been modified in the manuscript.

 3. Minor updates to the example PDF ( https://github.com/dmiller903/CompoundHetVIP/blob/master/CompoundHetVIP_example.pdf) were made for added clarity and explanation.
